# OP71 Patient Disease Strategy: A New Operational Framework For Collecting And Applying Patient Experience Data Into Clinical Development Programs

**DOI:** 10.1017/S0266462324001302

**Published:** 2025-01-07

**Authors:** Catherine Coulouvrat, Victoria DiBiaso, Stephanie Bascle, Laurence Lucats, Nathalie Largeron, Sophie Van Tomme, Benoit Arnould

## Abstract

**Introduction:**

Understanding patient experience and needs is crucial to develop high-value therapies. Patient experience data (PED) inform trial design and evidence generation plans. The U.S. Food and Drug Administration’s roadmap to patient-focused outcome measurement advocates integrating PED into product development. We adapted this theoretical framework to include the health technology assessment (HTA) perspective and operationalized it as a patient disease strategy (PDS) framework.

**Methods:**

The PDS framework is a methodology that systematically integrates patient-informed activities to reflect the patient health value of a new treatment. A PDS is developed per indication, initiated in the preclinical phase, applied in clinical development, and continuously adapted throughout the product development lifecycle. The three PDS phases include: (i) development of patient profile, including epidemiology, demographics, patient journey, disease, and treatment burden for patients and caregivers; (ii) PED gap analysis, focusing on identification of patient priorities, unmet needs, preferences, and expectations for new therapies; and (iii) translation into actions, such as diversity and inclusion (D&I) plans and outcomes strategy.

**Results:**

Out of 58 indications, 31 percent have endorsed PDS and 67 percent are in progress. Patient-relevant label opportunities increased by over 50 percent. Each indication was informed on average by patients from three different countries. The PDS framework helped to identify factors that impacted health outcomes for integration into trial designs and D&I plans. Early understanding of heterogeneous patient populations, unmet needs, benefit/risk trade-offs, and patient experiences ensured development programs measured the most meaningful outcomes while also addressing evidence gaps. Early understanding of patient priorities and barriers to participation optimized the studies by reducing burden and identifying proactive support needed to complete the trial.

**Conclusions:**

The PDS framework systematically identified health value opportunities for a target population and integrated the patient needs into the overall development plan. PED informs clinical trial design and endpoint strategy optimization, including factors that influence diversity and data integrity. We anticipate that the PDS will enable HTA decisions to reflect patients’ health value and ultimately improve access to innovative therapies.

